# Risk factors of metabolic dysfunction-associated fatty liver disease in Chinese nurses: an ambispective cohort study

**DOI:** 10.3389/fpubh.2025.1554793

**Published:** 2025-08-20

**Authors:** Jing Wang, Heli Zhang, Xiaotian Zhang, Jingpin Wang, Hongbo Chen, Baohua Li

**Affiliations:** ^1^Department of Medical Oncology and Radiation Sickness, Peking University Third Hospital, Beijing, China; ^2^Department of Rehabilitation Medicine, Peking University Third Hospital, Beijing, China; ^3^Nursing Department, Peking University Third Hospital, Beijing, China

**Keywords:** metabolic dysfunction-associated fatty liver disease, nurse, cohort study, risk factors, MAFLD

## Abstract

**Background:**

Metabolic dysfunction-associated fatty liver disease (MAFLD) refer to fatty liver disease related to systemic metabolic dysregulation, which is closely related to unhealthy lifestyles such as staying up late and eating irregularly. MAFLD has become most prevalent chronic liver disease and become a high incidence disease among nurses. Health and good condition of nurses are the basis to ensure the safety and quality of life of patients. Little is known about the risk factors of MAFLD in nurses.

**Method:**

We conducted an ambispective cohort study of the National Nurses' Health Study from 2018 to 2022 in a tertiary hospital in China. The data were collected by questionnaires and physical examination records. Analysis was done using SPSS 26.0. Risk factors for MAFLD were estimated by multivariable Cox proportional hazard regression using forward stepwise selection.

**Result:**

A total of 777 nurses were included in this study. The incidence of MAFLD in nurses exceeds the global average. Age at diagnosis (*p* = 0.011), BMI (*p* = 0.000), FBG (*p* = 0.048), TG (*p* = 0.009), uric acid (*p* = 0.011), female (*p* = 0.012), like eating oily food (*p* = 0.049) and spicy food (*p* = 0.028), and frequency of outgoing-food (*p* = 0.042) were risk factors for MAFLD.

**Conclusion:**

The incidence of MAFLD in nurses was higher than the global average and has become an occupational health concern. Age, BMI, female gender, fasting blood glucose (FBG), triglycerides (TG), uric acid, oily foods, spicy foods, and the frequency of eating out were risk factors for MAFLD occurrence. In the future, the focus should be on risk factors for MAFLD in nurses and developing intervention programs to improve nurse health and well being.

## Introduction

Metabolic dysfunction-associated fatty liver disease (MAFLD) refers to fatty liver disease linked to systemic metabolic dysregulation, evolving from non-alcoholic fatty liver disease (1). MAFLD has emerged as the most prevalent chronic liver disease globally. The global prevalence of MAFLD is estimated at 24%, with the highest rates in South America (31%) and the Middle East (32%), followed by Asia (27%) (2). Due to rapid urbanization, the prevalence and mortality rate of MAFLD in China have surged. In 2016, Chinese incidence rate of MAFLD surpassed that of the United States and European countries (3). If this trend continues, China will lead the world in both incidence and mortality rates of MAFLD (4). Initially asymptomatic, MAFLD can progress to advanced liver disease, cirrhosis, and hepatocellular carcinoma (5, 6). Additionally, MAFLD is associated with extrahepatic conditions like chronic kidney disease, inflammatory bowel disease, cardiovascular disease, and sleep apnea (7–9). A Chinese cohort study revealed that MAFLD patients face a higher risk of extra-hepatic cancers, such as thyroid and lung cancers, particularly among men (9, 10). Moreover, the prevalence of MAFLD is trending younger, posing serious threats to human health and societal development (4, 11).

MAFLD is influenced by various factors, including age, gender, race, body mass index, lipid metabolism index, and uric acid (12–14). With the rapid development of the global economy, increasing studies have revealed that MAFLD is related to health behavior (15). Obesity and overnutrition, driven by physical inactivity, sedentary lifestyle, diet, and sleep patterns, are strongly associated with MAFLD (16). As these lifestyle factors improved, the multivariable hazard ratio for MAFLD gradually decreased (17). In addition, night shifts, working long hours and high-intensity work increased the incidence of MAFLD (18–20).

Nurses' health is threatened by unhealthy lifestyles such as work pressure, bad eating habits and shift work, and MAFLD has become a high incidence disease for nurses (21). The health and well being of the nurse is the basis for ensuring patient safety and quality of life. However, previous studies have paid less attention to MAFLD in nurses, and most of the studies are cross-sectional and lack of research results on long-term habits, so it is difficult to accurately understand the risk factors of MAFLD in nurses' career development. Therefore, our study collected health status and lifestyle of nurses in Chinese hospitals from 2018 to 2022, to conduct a cohort analysis on MAFLD in Chinese nurses.

## Methods

### Study design and participants

This study was based on the National Nurse Health Study (NNHS), which was an ambispective cohort that collected physical examination results and web-based information on early-life events, daily habits, occupational and environmental risk factors, and health outcomes from a specific subset of healthcare professionals comprising Chinese nurses (22). We consecutively enrolled nurses and collected routine physical examination results annually starting in 2018 (Baseline). This study was registered on Clinicaltrials.gov (https://clinicaltrials.gov/ct2/show/NCT04572347) and the China Cohort Consortium (http://chinacohort.bjmu.edu.cn/project/102/) in cooperation with other hospitals. The study was approved by the ethics committee of Peking University Third Hospital. All participants gave written informed consent when they completed the baseline survey.

This study included nurses registered at Peking University Third Hospital who regularly participated in routine physical examinations. We excluded participants with the following characteristics: history of MAFLD, history of drinking alcohol and pregnancy. Initially, a total of 1,335 individuals were enrolled in the NNHS nurses underwent examinations between January 2018 and December 2022 at Peking University Third Hospital in Beijing, China (22).

### Data collection

We used data collected from annual worksite health visits, during which employees in the hospital receive regular physical examinations. Any nurse who is unable to participate at the regularly scheduled physical examination may participate in a supplementary examination. All relevant data were abstracted from the Tianrui Kangjian Information System (Tianrui Kangjian (Fuzhou) Information Technology Co., Ltd., Fujian, China). The information center regularly maintained the database to ensure its security. Health examination data were exported with the assistance of the information center in text form with all identification information hidden. The database included physical measurements (i.e., height, weight, blood pressure (BP), and pulse rate) and a biochemical profile [i.e., fasting blood glucose (FBG), cholesterol, triglyceride (TG), aspartate, uric acid, high-density lipoprotein cholesterol (HDL-C), and low-density lipoprotein cholesterol (LDL-C)].

We used a self-designed questionnaire to inquire about nurses' work and dietary habits. The questionnaire included four items, including whether to exercise regularly (≥3 times/week), whether night-shifts, taste preferences (light, salty, spicy, and oily), and frequency of dining-out (specifically refers to food delivered by delivery personnel). Specific examples (e.g., spicy food was perceived as hotpot, mapo tofu, chili stir-fries) were provided in the questionnaire to ensure consistent interpretation. All variables that we used prospectively collected data during follow-up visits to validate baseline retrospective reports.

All procedures followed pre-defined, validated protocols (e.g., standardized questionnaires, calibrated instruments). And field researchers received comprehensive training. Electronic data capture systems enforced real-time validation (e.g., range checks, logic constraints, mandatory fields). Also an independent statistician reviewed 100% of primary endpoint data prior to analysis.

### Data measurement

Demographic information and history were recorded using web-based data. Eligible participants logged onto the online survey system using their name and nurse registration number. Height and weight were measured using a regularly corrected height-weight scale (HNH-318, Omron Co., Ltd, Ch, Japan). Systolic and diastolic BP (SBP and DBP, respectively) and pulse rate were measured using an automatic sphygmomanometer (HBP 9021, Omron Co., Ltd, Ch, Japan) and recorded after the individual had rested for at least 5 min. BMI was calculated by dividing weight (in kilograms) by height (in square meters).

Biochemical profiles were measured from venous blood samples between 7:00 and 9:00 a.m. after an overnight fast of 8–12 h. Serum biochemical markers were measured using an AU5800 automated analyzer (Beckman Coulter Life Sciences, USA).

Table 1 shows a list of laboratory values and reference values. Liver ultrasonography was performed by radiologists using a color Doppler ultrasound diagnostic apparatus (S1GKM3HKA00004Z, RS80A, South Korea) for liver examination. The diagnosis of MAFLD, gallstone and thyroid nodule were obtained from a review of radiological reports and daily life habits. MAFLD was diagnosed by a physician and endocrinologists.

**Table 1:** Laboratory values.

**Domain**	**Parameter**	**Instrument/technique**	**Assessing person**	**Reference value**
Demographic characteristics	1. Marital status 2. Gender	Self-administered web-based questionnaire	Participant	–
Health status	1. History and current illness	Self-administered web-based questionnaire	Participant	–
Dietary preferences	1. Favorite food taste	Self-administered web-based questionnaire	Participant	–
	2. Frequency of outgoing-food			Times/month
Anthropometry	1. Height	Physical examination	Nurses	-cm
	2. Weight			-kg
	3. Body Mass Index (BMI)			18.5–24 kg/m^2^
	4. Blood pressure			90–139/60–89 mmHg
	5. Heart rate			60–100/min
Laboratory test	1. Total cholesterol (TC)	Physical examination	Nurses	< 5.8 mmol/L
	2. Triglyceride (TG)			< 1.7 mmol/L
	3. High-density lipoprotein cholesterol (HDL-C)			>1.04 mmol/L
	4. Low density lipoprotein cholesterol (LDL-C)			< 3.64 mmol/L
	5. Fasting blood glucose (FBG)			3.9–6.1 mmol/L
	6. Uric acid			208–428 μmol/L
	7. Alanine aminotransferase (ALT)			7–40 U/L
	8. Aspartate aminotransferase (AST)			13–35 U/L
	9. White blood cell (WBC)			3.5–9.5 × 10^9^/L
	10. Red blood cell (RBC)			3.8–5.1 × 10^9^/L
	11. Platelet (PLT)			125–350 × 10^9^/L

Employee numbers were used to directly extract physical examination data and laboratory test results from the system. Each participant took part in a comprehensive physical examination at baseline and at 1-year intervals. All identification numbers in the database were encrypted to ensure the privacy of each individual.

### Statistical analysis

Statistical analysis was performed with SPSS 26.0 (SPSS Inc., Chicago, IL, USA). In all cases, differences for which *p* < 0.05 were considered statistically significant; all *p* values were two-sided. Because the data samples are basically normally distributed, and the missing data is < 3% of the overall data. Normally distributed data are expressed as mean ± standard deviation. Variables with non-normal distributions are expressed as the median (interquartile range) and were compared between two groups by the Mann–Whitney *U* test. Risk factors for MAFLD were estimated by multivariable Cox proportional hazard regression using forward stepwise selection. Removal testing was based on the probability of the Wald statistic.

## Results

### Characteristics of the study population

**Figure 1:**
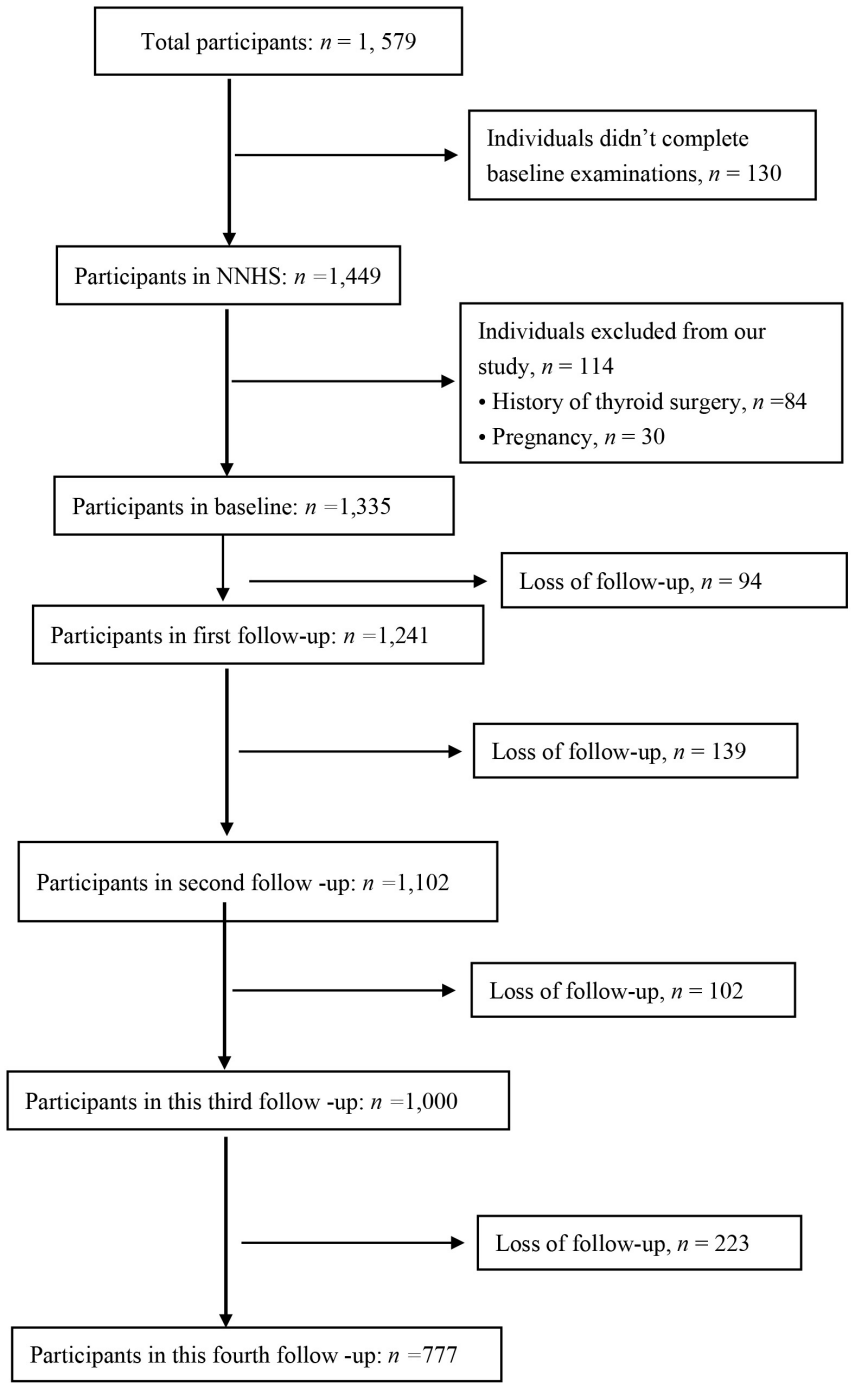
Participants' flow diagram.

We selected 1,335 individuals without missing data at baseline (2018) for evaluation. A total of 558 nurses were excluded due to resignation, retirement or pregnancy at the third follow-up. Subsequently, 777 nurses were included in analysis (Figure 1). Participants were informed of the purpose and procedures of the NNHS.

The nurses in this study were 31.96 ± 6.85 years old. The BMI of the nurses was 22.31 ± 2.74 kg/m^2^. The general characteristics and laboratory results of the participants are shown in Table 2. While, Table 3 shows the working and dietary conditions of nurses.

**Table 2:** The general characteristics and laboratory results of participants.

**Variables**	**Baseline-2018 (*n* = 1,335)**	**Follow-up 1-2019 (*n* = 1,241)**	**Follow-up 2-2020 (*n* = 1,102)**	**Follow-up 3-2021 (*n* = 1,000)**	**Follow-up 4-2022 (*n* = 777)**
Age (years)	31.96 ± 6.85 (20.00–53.00)	33.25 ± 6.86 (21.00–54.00)	34.34 ± 7.04 (22.00–55.00)	35.23 ± 5.06 (23.00–56.00)	36.40 ± 6.81 (25.00–57.00)
BMI (kg/m^2^)	22.31 ± 2.74 (16.00–39.00)	22.37 ± 2.88 (15.73–34.63)	22.35 ± 2.75 (16.00–24.10)	22.41 ± 3.02 (15.96–34.99)	22.64 ± 3.07 (15.80–34.36)
**Gender**					
Female	1,271 (95.2)0	1,182 (95.20)	1,052 (95.50)	957 (95.70)	744 (95.80)
Male	64 (4.80)	59 (4.80)	50 (4.50)	43 (4.30)	33 (4.20)
**Marital status**					
Married	883 (66.10)	865 (69.70)	795 (72.10)	759 (75.9)	595 (76.60)
Unmarried	452 (33.80)	376 (30.30)	307 (17.90)	241 (24.10)	182 (23.40)
History of hypertension	13 (1.00)	16 (1.30)	15 (1.40)	16 (1.60)	17 (2.19)
History of diabetes	3 (0.20)	2 (0.20)	1 (0.10)	4 (0.40)	3 (0.39)
History of hyperlipidemia	1 (0.10)	1 (0.10)	12 (1.10)	12 (1.20)	10 (1.29)
Gallstone	28 (2.20)	8 (0.60)	23 (2.10)	23 (2.30)	2 (0.26)
Thyroid nodule	586 (44.90)	529 (42.60)	332 (30.10)	315 (31.50)	367 (47.23)
SBP (mmHg)	116.38 ± 11.55 (80.00–165.00)	118.02 ± 10.97 (84.00–173.00)	119.87 ± 10.88 (86.00–160.00)	119.38 ± 11.54 (91.00–166.00)	120.01 ± 12.04 (86.00–169.00)
DBP (mmHg)	70.06 ± 8.70 (42.00–102.00)	72.87 ± 8.60 (46.00–116.00)	73.52 ± 8.54 (43.00–109.00)	72.91 ± 8.96 (46.00–114.00)	72.61 ± 8.91 (53.00–124.00)
Fasting blood glucose	4.78 ± 0.50 (2.50–10.09)	4.65 ± 0.63 (3.00–15.10)	4.98 ± 0.53 (3.60–13.00)	5.22 ± 0.52 (3.70–10.40)	5.26 ± 0.54 (4.00–11.60)
Pulse rate	82.53 ± 10.51 (52.00–131.00)	83.08 ± 10.24 (55.00–127.00)	82.40 ± 10.43 (51.00–123.00)	82.06 ± 10.81 (51.00–117.00)	83.13 ± 10.71 (53.00–124.00)
Cholesterol	4.43 ± 0.76 (2.38–8.37)	4.54 ± 0.75 (2.58–8.69)	4.63 ± 0.78 (2.45–10.03)	5.01 ± 0.86 (2.98–8.64)	4.81 ± 0.82 (2.73–8.01)
HDL-C	1.53 ± 0.30 (0.76–2.73)	1.47 ± 0.28 (0.75–2.73)	1.48 ± 0.29 (0.77–2.80)	1.51 ± 0.31 (0.67–2.85)	1.45 ± 0.31 (0.76–2.53)
LDL-C	2.64 ± 0.65 (0.57–6.32)	2.78 ± 0.64 (0.66–6.37)	2.94 ± 0.67 (0.93–6.63)	2.77 ± 0.64 (0.65–5.97)	2.75 ± 0.65 (0.85–5.51)
Triglycerides	0.91 ± 0.04 (0.29–5.98)	1.99 ± 0.63 (0.28–13.82)	0.97 ± 0.05 (0.35–10.08)	0.93 ± 0.05 (0.31–4.82)	1.03 ± 0.54 (0.21–5.18)
Uric acid	262.91 ± 56.09 (127.00–576.00)	273.69 ± 59.53 (104.00–579.00)	262.61 ± 55.83 (137.00–512.00)	264.87 ± 60.56 (115.00–577.00)	272.00 ± 61.22 (145.00–587.00)
ALT	15.42 ± 10.90 (4.00–182.00)	15.20 ± 1.70 (4.00–175.00)	15.64 ± 9.09 (4.00–93.00)	20.00 ± 7.13 (6.00–85.00)	21.19 ± 8.38 (9.00–92.00)
AST	18.05 ± 6.60 (9.00–122.00)	18.49 ± 7.17 (10.00–152.00)	18.93 ± 5.60 (10.00–87.00)	17.93 ± 12.76 (5.00–116.00)	20.62 ± 7.35 (10.00–79.00)
WBC	6.09 ± 1.58 (2.51–16.82)	6.04 ± 1.46 (1.93–14.42)	6.03 ± 1.49 (2.41–11.77)	5.94 ± 1.52 (2.55–16.57)	6.14 ± 1.67 (2.66–16.24)
RBC	4.47 ± 0.35 (3.35–5.86)	4.50 ± 0.35 (3.40–6.13)	4.44 ± 0.35 (3.51–5.83)	4.47 ± 0.36 (3.35–6.04)	4.41 ± 0.34 (3.28–5.91)
PLT	260.29 ± 56.09 (90.00–609.00)	262.90 ± 55.87 (89.00–554.00)	260.02 ± 55.88 (90.00–548.00)	260.87 ± 55.82 (93.00–532.00)	265.38 ± 60.31 (87.00–509.00)

BMI, Body Mass Index; SBP, Systolic Blood Pressure; DBP, Diastolic Blood Pressure; HDL-C, High-density lipoprotein cholesterol; LDL-C, Low density lipoprotein cholesterol; ALT, Alanine aminotransferase; AST, Aspartate aminotransferase; WBC, White blood cell; RBC, Red blood cell; PLT, Platelet.

**Table 3:** Working and dietary conditions of nurses.

**Items**	***N* (%) (*n* = 777)**
**Exercise**	
Yes	250 (32.20)
No	527 (67.80)
**Favorite food taste**	
Light	361 (46.50)
Salty	127 (16.30)
Spicy	257 (33.10)
Oily	32 (4.10)
**Night shifts**	
Yes	410 (52.80)
No	367 (47.20)
**Frequency of outgoing-food**	
1–4 times/month	66 (8.50)
5–10 times/month	338 (43.50)
1–4 times/week	108 (13.90)
>5 times/week	79 (10.20)
Everyday	196 (23.90)

### Incidence of MAFLD

**Figure 2:**
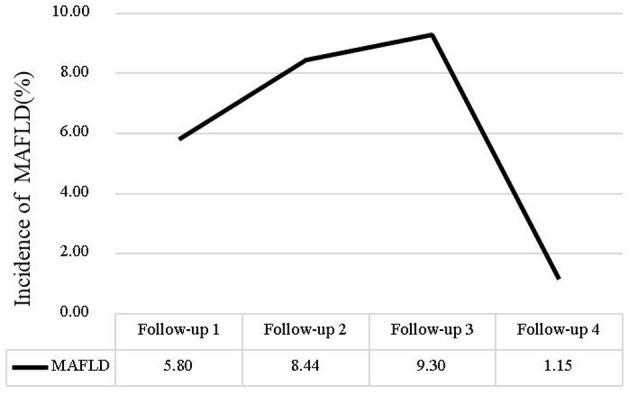
The incidence of various MAFLD.

We calculated MAFLD incidence using the number of nurses who newly diagnosed MAFLD each year/the number of total nurses that participated in the physical examinations each year. The incidence rate of MAFLD was 5.8% in 2019, 8.44% in 2020, 9.30% in 2021. However, in 2022 the incidence rate of MAFLD has decreased, the incidence rate of MAFLD was 1.15%. The change in the MAFLD incidence (%) rate over time is shown in Figure 2.

### Multivariate cox regression analysis of risk factors in MAFLD

In Cox regression model, age at diagnosis, BMI, TC, TG, HDL-C, LDL-C, and uric acid were included as continuous variables, whereas gender (1 = male; 2 = female), frequency of outgoing-food(), favorite food taste (1 = light, 2 =salty, 3 = spicy, 4 = oily), exercise (1 = yes, 0 = no), night shifts (1 = yes, 0 = no) and MAFLD were dichotomous variables (1 = presence of condition; 0 = absence of condition). Age at diagnosis (HR: 1.039; 95% CI, 1.009–1.070 *p* = 0.011), BMI (HR: 1.212; 95% CI, 1.133–1.297 *p* = 0.000), FBG (HR: 1.544; 95% CI, 1.004–2.406, *p* = 0.048), TG (HR: 2.110; 95% CI, 1.202–3.704, *p* = 0.009), uric acid (HR: 1.005; 95% CI, 1.001–1.009, *p* = 0.011), female (HR: 3.678; 95% CI, 1.335–10.129, *p* = 0.012), like eating oily food (HR: 1.826; 95% CI, 1.004–3.321, *p* = 0.049), and spicy food (HR: 1.720; 95% CI, 1.061–2.782, *p* = 0.028) and frequency of outgoing-food (HR: 1.850; 95% CI, 1.024–3.341, *p* = 0.042) were risk factors for MAFLD (Table 4).

**Table 4:** Multivariate cox regression analysis of risk factors in MAFLD.

**Variable**	**HR**	**95% CI**		***p-*Value**

		**Lower**	**Upper**	
Age	1.039	1.009	1.070	0.011
BMI	1.212	1.133	1.297	0.000
FBG	1.544	1.004	2.406	0.048
TG	2.110	1.202	3.704	0.009
Uric acid	1.005	1.001	1.009	0.011
Female	3.678	1.335	10.129	0.012
Like eating oily food	1.826	1.004	3.321	0.049
Like eating spicy food	1.720	1.061	2.782	0.028
Frequency of outgoing-food	1.850	1.024	3.341	0.042

BMI, body mass index; FBG, fasting blood glucose; TG, triglyceride.

## Discussion

Numerous studies have identified risk factors associated with MAFLD. However, due to variations in populations and methodologies, the findings are often inconsistent. This inconsistency is particularly evident in studies focusing on nurses and longitudinal cohort studies. This research investigated the risk factors for MAFLD among nurses over a long-term period, examining variables such as age, BMI, gender, fasting blood glucose, triglycerides, uric acid levels, consumption of oily and spicy foods, the frequency of dining out, night shifts and frequency of exercise.

The study found the incidence of MAFLD remained above 5% from 2018 to 2021, exceeding the global average (23). However, a sharp decline in incidence was observed in 2022, likely due to the limited study population and the high risk of MAFLD-associated nurses has already had between 2018 and 2021. And nurses with prior MAFLD diagnoses might have been lost to follow-up, as they were no longer included in “new case” counts. The “healthy worker effect” may be amplified in nursing populations, where occupational health screenings create earlier detection and intervention opportunities. Additionally, since MAFLD can be alleviated or even reversed through diet and exercise, some nurses may have prevented MAFLD through health behavior interventions (24, 25).

The study identified BMI as a risk factor for MAFLD in nurses, consistent with other research (13, 14). Notably, BMI's limitation in assessing body shape (26) may explain why about 25% of MAFLD patients (particularly Asians) are non-obese. Our findings confirm that combining BMI with ABSI significantly improves MAFLD risk prediction over BMI alone (27), supporting waist circumference as a valuable obesity-related MAFLD predictor. MAFLD risk increased with age in our cohort, mirroring trends in younger Chinese populations (14). Since participants were young, this contrasts with studies of general populations or retirees (15). While most studies report higher female MAFLD risk (11, 28), some indicate male predominance, potentially due to estrogen deficiency exacerbating liver inflammation (14).

We discovered that fasting blood glucose, triglycerides, and uric acid are independent risk factors for MAFLD, and these factors are interrelated (13, 14). MAFLD, a lipid metabolism disorder, results in fat accumulation in liver cells and is associated with insulin resistance and abnormal blood glucose levels. This condition hampers the oxidative breakdown and utilization of free fatty acids in the liver, leading to increased triglyceride production (29–31). Elevated blood uric acid levels can exacerbate insulin resistance, thereby heightening the risk of MAFLD (32). Our findings align with previous research.

The study identified a correlation between MAFLD and the frequency of consuming outgoing foods, characterized by greasy and highly processed items. A meta-analysis revealed that high intakes of processed foods, red meat, high-fat dairy products, and refined grains significantly elevated the risk of MAFLD (16). Conversely, the Mediterranean diet (MD), abundant in polyunsaturated fats, polyphenols, vitamins, and carotenoids—found in oily fish/fish oil, coffee, nuts, vegetables, and whole grains—exhibited protective effects (16, 33). Omega-3 supplementation might reduce the risk of liver disease, particularly non-alcoholic liver disease. In this study, spicy foods are defined by the presence of chili as the main spice (34). Although limited research exists on the direct link between spicy foods and MAFLD risk, such foods may negatively impact overweight/obesity and thereby indirectly contribute to MAFLD (35, 36). Studies suggest that spicy foods may increase the consumption of carbohydrates, heavily salted or oily meats, and sweet foods to mitigate the spicy sensation. Additionally, with chili consumption exceeding 50 g/day, capsaicin can desensitize vagal nerve events, reducing satiety signals and increasing overall food intake (37, 38).

Contrary to numerous previous studies, our research indicated that neither engaging in physical exercise nor night shift were significant risk factors for MAFLD among the Chinese nurse population investigated (39–41). This discrepancy prompts a detailed exploration of potential reasons, informed by the existing literature and the study design. Previous studies have highlighted the adverse effects of shift work on disrupting circadian rhythms and causing metabolic disorders, particularly in women and older individuals (20). But the study did not address the content and intensity of shift work. Night shift nurses are required to maintain a high degree of physical activity due to frequent patient visits, nursing procedures, and emergency handling. We speculate that this sustained moderate-intensity physical activity during the night shift may play a key role in counteracting the potential metabolic risks associated with the night shift. Therefore, the results of this study indicate that the hazards of night shifts can be greatly reduced or even completely eliminated by high-intensity work. Future research needs to consider the importance of “work patterns” rather than simply categorizing “whether or not to work night shifts” as a binary classification.

## Limitation

The study has some unavoidable limitations. Firstly, while a multi-center study was preferred, data collection proved too challenging, resulting in data being gathered from a single hospital. Secondly, the retrospective nature of data collection through questionnaires might introduce bias. Lastly, the study's predominantly female population may affect the generalizability of the results, particularly the risk factors for fatty liver in male nurses. Future studies should prioritize multicenter collaborations involving diverse healthcare settings to enhance the external validity and broader applicability of findings. Additionally, concerted efforts are needed to recruit more balanced cohorts in terms of gender to elucidate potential sex-specific differences in risk factors of MAFLD.

## Conclusion and implications

As health managers, nurses' personal health significantly impacts the quality of nursing care and patient safety. This study may be the first long-term cohort study examining risk factors for MAFLD in nurses. Identified risk factors include age, BMI, female gender, fasting blood glucose, triglycerides, uric acid, oily foods, spicy foods, and the frequency of eating out. Future efforts should focus on developing comprehensive treatment and prevention programs that address these risk factors, specifically promoting healthier dietary choices when eating out (e.g., Mediterranean-style meals, reduced spicy foods, refined carbs, and red meat), encouraging regular physical activity and weight management, and monitoring lipid metabolism, thereby enhancing the health of healthcare managers.

## Data Availability

The raw data supporting the conclusions of this article will be made available by the authors, without undue reservation.
